# Intraspecific Variation in Cellular and Biochemical Heat Response Strategies of Mediterranean *Xeropicta derbentina* [Pulmonata, Hygromiidae]

**DOI:** 10.1371/journal.pone.0086613

**Published:** 2014-01-27

**Authors:** Sandra Troschinski, Maddalena A. Di Lellis, Sergej Sereda, Torsten Hauffe, Thomas Wilke, Rita Triebskorn, Heinz-R. Köhler

**Affiliations:** 1 Animal Physiological Ecology, Institute of Evolution and Ecology, Tübingen University, Tübingen, Germany; 2 Department of Animal Ecology & Systematics, Justus Liebig University Giessen, Giessen, Germany; Institution and Department: Agricultural Research Service, United States of America

## Abstract

Dry and hot environments challenge the survival of terrestrial snails. To minimize overheating and desiccation, physiological and biochemical adaptations are of high importance for these animals. In the present study, seven populations of the Mediterranean land snail species *Xeropicta derbentina* were sampled from their natural habitat in order to investigate the intraspecific variation of cellular and biochemical mechanisms, which are assigned to contribute to heat resistance. Furthermore, we tested whether genetic parameters are correlated with these physiological heat stress response patterns. Specimens of each population were individually exposed to elevated temperatures (25 to 52°C) for 8 h in the laboratory. After exposure, the health condition of the snails' hepatopancreas was examined by means of qualitative description and semi-quantitative assessment of histopathological effects. In addition, the heat-shock protein 70 level (Hsp70) was determined. Generally, calcium cells of the hepatopancreas were more heat resistant than digestive cells - this phenomenon was associated with elevated Hsp70 levels at 40°C.We observed considerable variation in the snails' heat response strategy: Individuals from three populations invested much energy in producing a highly elevated Hsp70 level, whereas three other populations invested energy in moderate stress protein levels - both strategies were in association with cellular functionality. Furthermore, one population kept cellular condition stable despite a low Hsp70 level until 40°C exposure, whereas prominent cellular reactions were observed above this thermal limit. Genetic diversity (mitochondrial cytochrome c oxidase subunit I gene) within populations was low. Nevertheless, when using genetic indices as explanatory variables in a multivariate regression tree (MRT) analysis, population structure explained mean differences in cellular and biochemical heat stress responses, especially in the group exposed to 40°C. Our study showed that, even in similar habitats within a close range, populations of the same species use different stress response strategies that all rendered survival possible.

## Introduction

Animals which live in dry and hot habitats have to cope with desiccation and overheating. Land snails are particularly affected by these adverse conditions due to their water-permeable skin [1] and, therefore, developed a range of behavioral, physiological, and morphological adaptations to ensure survival in arid habitats. Climbing on vegetation to escape from hot ground temperature and restriction of activity phases to favorable time periods can be regarded as behavioral adaptations [2], [3]. As a physiological mechanism of adaptation, aestivation accompanied by a decrease of the metabolic rate [4], [5] during dry and hot seasons allows land snails even to survive in extreme arid climates of deserts [6]. Morphological adaptations are reflected in variations in shell structure, shell aperture, and size, as well as in body and shell color, or the thickness of the epiphragm [3], [7], [8].

The hepatopancreas plays a major role in the metabolism of mollusks [9], [10]. Alterations and cellular damage caused by different stressors, including heat stress, rapidly occur in the hepatopancreas, which makes this organ suitable to monitor and study cellular responses [11], [12]. It is also known that calcium cells, representing one cell type of the hepatopancreas, play an important role in osmoregulation [10] and acid-base balance [13], both of which can be affected by high temperature.

Stress or heat shock proteins (Hsp) can be induced by heat and many other proteotoxic stressors in several organisms [14], [15]. The 70 kD stress protein family, Hsp70, is a main component of the cellular heat stress response system and protects the cell against the proteotoxic action of elevated temperature and numerous other stressors [16]–[18]. Though isoforms of this protein class are constitutively present already under homeostatic conditions in the cell - e.g. acting as chaperones during protein folding processes, stabilizing proteins in intracellular trafficking, and playing an essential role in assembly, degradation and intracellular localization of proteins [19]–[21] - the expression of some isoforms of Hsp70 is up-regulated under the influence of a proteotoxic stressor and, therefore, can be used as a marker for proteotoxic stress. Moreover, several studies indicate that genetic differences among populations may be associated with Hsp70 expression levels [22]–[24], though the effect of population structure on heat response differences in invertebrates is, in general, not well understood.

A land snail species that seems to be particularly well adapted to heat stress is the pulmonate *Xeropicta derbentina* (Krynicki, 1836). These snails are highly abundant in the Mediterranean region and can build up large populations with hundreds of thousands of individuals. During daytime, they remain inactive on vegetation, fully exposed to sunlight. Although several studies exist on thermotolerance and adaptations to heat stress in land snails in general [3], [25], [26] and in Mediterranean species in particular [27]–[31], as well as on the genetic structure in the Helicidae [32]–[34], comprehensive investigations combining cellular and biochemical reactions to heat stress with population structure information are lacking. We here used *X. derbentina* as a model organism and investigated variations in heat response mechanisms after exposure to elevated temperatures in seven populations.

Specifically, we studied whether different populations of the same species collected within a range of a few kilometers in similar habitats utilize different biochemical and cellular strategies to deal with heat stress, and tested whether physiological heat stress response data can be explained with population structure information. For this purpose, genetically characterized specimens were individually exposed to elevated temperatures (25 to 52°C) under laboratory conditions. Then, effects of heat stress were assessed by histopathological (cellular) and stress protein level (biochemical) biomarkers, and the correlation between genetic structure and heat stress response data tested using multivariate statistics.

## Materials and Methods

### Ethics statement

No specific permissions for sampling were required for public land in which some of the snails were sampled. Part of the samples derived from private land the owner of those has given permission. Sampling took place according to the directions of the Convention of Biological Diversity. The study did not involve protected or endangered species.

### Sampling sites

Individuals of seven populations of *Xeropicta derbentina* were collected in the last week of May 2010 in the Vaucluse area, Provence, Southern France (Table 1). All sampling sites were dry, open, and sun-exposed habitats and similar in structure and vegetation.

**Table 1:** Coordinates and locality of the different sampling sites.

Population	Locality	Coordinates
1	Modène 1	N 44° 6.055′ E 5° 7.937′
2	Modène 2	N 44° 6.157′ E 5° 7.733′
3	Modène 3	N 44° 6.391′ E 5° 7.032′
4	St. Pièrre	N 44° 6.053′ E 5° 8.311′
5	Mazan 1	N 44° 1.511′ E 5° 6.446′
6	Mazan, Bon Reméde	N 44° 2.653′ E 5° 8.213′
7	Mazan 2	N 44° 3.974′ E 5° 8.084′

For each sampling site, approximately 200 snails were collected and kept separately in plastic containers (20.5×30×19.5 cm). For genetic analyses, 20 snails per sampling site were collected and stored in liquid nitrogen.

### Experimental setup

In the laboratory, the snails were acclimatized to 25°C for 2 weeks. The plastic containers were filled with a layer of ground-cover material for terrariums (JBL, Terra Basis, Neuhofen, Germany). The snails were fed organic milk mash (Hipp, Pfaffenhofen, Germany) *ad libitum* and sprayed with water two times per week to assure an appropriate level of humidity.

The temperature experiments were conducted in heating cabinets using smaller plastic boxes (6.5×18×13 cm) lined with moist paper towels and covered with perforated plastic sheets. Twenty-two individuals per population were exposed as a group in individual plastic containers to temperatures of 25, 33, 38, 40, 43, 45, 48, 50 and 52°C for 8 h. As the two highest temperature regimes (50 and 52°C) were lethal for the snails, these groups were excluded from both histopathological and stress protein analyses. Due to constraints in lab capacity, snails exposed to 38 and 45°C were not investigated histologically. 25°C was used as control temperature.

Even though tub effects cannot be totally excluded in such an experimental design, we adjusted the conditions in the different plastic boxes equal, to the best of our possibilities, in order to minimize them.

After eight hours of exposure, eight randomly selected individuals from each experimental group were used for the histological studies. For the stress-protein analyses, ten individuals per group were individually shock-frozen in liquid nitrogen and stored at −25°C until further analysis. In order to avoid a potential bias introduced by body size, we conducted a pre-test correlating Hsp70 values (both base level and levels recorded after temperature exposure) and body size (sliding caliper measured shell diameter).

### Histopathological analyses

First, the shells of the snails were cracked between two glass slides and removed. Immediately after cracking, the snails were fixed in 2% glutardialdehyde (25% glutardialdehyde dissolved in 0.01 M cacodylate buffer, pH 7.4) and stored for at least one week at 4°C. After overnight de-calcification in a 1∶2 mixture of formic acid and ethanol (70%) to remove leftover shell fragments, the samples were dehydrated in a graded series of ethanol and embedded in epoxy resin (Technovit, Heraeus Kulzer GmbH, Wehrheim, Germany). Tissue sections with a thickness of 7 µm were prepared using a Reichert Jung 2050 rotation microtome, stained with haematoxylin-eosin, and analyzed by light microscopy.

For each individual, the condition of the hepatopancreas cells (digestive and calcium cells only; excretory cells were excluded from the analysis) and the structural appearance of the tubules were qualitatively described and semi-quantitatively assessed according to the method described by Dittbrenner et al. [27]. For the semi-quantitative assessment, five categories at a scale from 1 to 5 reflecting the histopathological damage were defined: category 1, control status; category 3, status of reaction; category 5, status of destruction; categories 2 and 4 are chosen as intermediate stages between 1 and 3 or 3 and 5, respectively. Table 2 shows the criteria for each cell type and the tubule tissue for the classification into the three main categories.

**Table 2:** Criteria for histopathological effects in the tubule and cell types in the hepatopancreas for classification in the three main categories of the semi-quantitative assessment.

	Category 1: control status	Category 3: status of reaction	Category 5: status of destruction
Digestive cells	Columnar in shape	Irregular cell shape	• Cells damaged
	Nucleus oval in shape	Irregular nucleus shape	• Necrosis
	Clear cellular compartmentation (vacuolisation: apical small, basal large vacuoles)	Irregular cellular compartmentation (irregular vacuolisation)	
Calcium cells	Cone-shaped cells (broad basis, slim apex)	Irregular cell shape	• Cells damaged
	Large round-shaped nucleus	Irregular nucleus shape	• Necrosis
	Dense and consistent cytoplasm	Irregular cytoplasm	
Tubule	Smooth apices	Irregular apices	• Tubule damaged
	Smooth basic	Large lumina	
	Tight lumina		

The condition of the tubules and the two cell types were individually assessed for each snail and, finally, the individual assessments were averaged to get a mean assessment value (MAV) for each population at a given temperature. In addition, the average percentage of calcium cells in the hepatopancreas was determined according to the method by Dittbrenner et al. [27].

### Stress protein analyses (HSP70)

Deep-frozen individuals were homogenized on ice in extraction buffer (80 mM potassium acetate, 5 mM magnesium acetate, 20 mM Hepes and 2% protease inhibitor at pH 7.5) according to their body mass (2 µL buffer/mg snail) and centrifuged for 10 minutes at 20,000 *g* and 4°C. To determine the total protein content of each sample, the method of Bradford [35] via protein-dye binding assay was used. Constant protein weights (40 µg per sample) were separated by minigel SDS-PAGE (12% acrylamide, 0.12% bisacrylamide, 30 minutes at 80 V, and 75–90 minutes at 120 V) and transferred to nitrocellulose membranes by semi-dry Western blotting. The membranes were blocked in a 1∶2 mixture of horse serum and TBS (50 mM Tris, pH 5.7, 150 mM NaCl) for 2 hours. Subsequently, the membranes were incubated in the first antibody solution containing monoclonal α-Hsp70 antibody (mouse anti-human Hsp70, Dianova, Hamburg, Germany, dilution 1∶5000 in 10% horse serum in TBS) on the lab shaker at room temperature overnight. After washing for 5 minutes in TBS, membranes were incubated in the second antibody solution (goat anti-mouse IgG conjugated to peroxidase, Jackson Immunoresearch, West Grove, PA, dilution 1∶1000 in 10% horse serum/TBS) on a lab shaker for 2 hours at room temperature. Following another washing step in TBS, the developed antibody complex was detected by staining with a solution of 1 mM 4-chloro(1)naphthol, 0.015% H_2_O_2_, 30 mM Tris pH 8.5, and 6% methanol. The optical volume (area of the bands [number of pixels] × average grey scale value after background subtraction) of the Western blot protein bands was quantified using a densitometric image analysis system (E.A.S.Y. Win 32, Herolab, Wiesloch, Germany). For each sample, data were related to an internal Hsp70 standard (extracted from *Theba pisana* snails) to assure comparability.

For each population, the maximum percentage of stress protein (Hsp70) induction was determined as the quotient of the Hsp70 level for the respective exposure groups and the Hsp70 level of the control group at 25°C (control = 100%).

### DNA isolation, amplification and sequencing

Genomic DNA was extracted from the foot tissue of deep-frozen specimens using the DNeasy Blood & Tissue Kit (QIAGEN, Inc., Mississauga, Ontario, USA). We amplified a fragment of the mitochondrial cytochrome *c* oxidase subunit I (COI) gene with a target length of 700 base pairs (excluding primer sequence). Forward and reverse primers for PCR amplification and DNA sequencing were LCO1490 [36] and the newly developed primer HeliR2 5′-CCTAAAATATGWGAAAYAATACCAAA-3′. Bidirectional DNA sequencing according to the ‘Sanger’ chain-termination method was performed by LGC Genomics (Berlin, Germany) using an ABI 3730 XL DNA analyzer. Consensus sequences were generated in BioEdit 7.0.9.0 [37] and deposited in GenBank (GenBank accession numbers KF734452- KF734589).

### Statistical analyses

#### Correlation analyses of histopathological and stress protein data

Histopathological and biochemical (Hsp70) data were analyzed using JMP 9 (SAS Institute Inc., Cary, NC). As the Shapiro-Wilk test showed data not to be normally distributed, the nonparametric Wilcoxon U-Test was used to detect significant differences between the control group and each treatment. To counteract the problem of multiple comparisons, a Bonferroni correction was used. For histopathological data, the levels of significance were defined as: 0.0025<*P*≤0.0125: * (slightly significant); 0.00025<*P*≤0.0025: ** (significant); *P*≤0.00025: *** (highly significant). For the data of the stress protein induction (Hsp70 level), the levels of significance were defined as: 0.0017<*P*≤0.0083: * (slightly significant); 0.00017<*P*≤0.0017: ** (significant); *P*≤0.00017: *** (highly significant) after Bonferroni correction.

Correlations of relative Hsp70 levels vs. snail shell sizes were conducted using JMP 9. Correlations of the relative Hsp70 levels vs. the histopathological assessment values recorded for the respective cell types and the tubule condition as well as the illustration of the ratio of digestive cell/calcium cell integrity vs. temperature and the correlation of relative Hsp70 level vs. histopathological condition illustrating the population's heat response strategies were done with SigmaPlot 2000 (SPSS Inc.).

Analysis was conducted with JMP 9. Based on the results for Hsp70 levels which revealed considerable differences in the populations' maximum of stress protein induction at 40°, we used the Hsp70 response at this temperature to test for statistically significant differences among populations. The separate analysis of the factors was necessary since they showed a significant interaction (2-way ANOVA, F = 4.411, p<0.0001). Normality and homogeneity of variance were confirmed with the Shapiro-Wilk test (W = 0.9791; p = 0.2923) and the Levene's test (F_6,63_ = 1.8059; p = 0.1122). To detect significant differences among populations in this treatment, we used an ANOVA followed by the Tukey-Kramer HSD post-hoc test. Level of significance was set to 0.05. The box plot illustration was done with SigmaPlot 2000.

#### Network analysis

Cryptic species may coexist within the range of morphologically undistinguishable heliciid snails [38]. For excluding this possibility for our sample populations of *X. derbentina*, we constructed a statistical parsimony haplotype network from all sequences generated in order to test whether all haplotypes can be connected in a parsimonious fashion. The analysis was done using the program TCS 1.21 [39] with the default connection limit of 95%.

#### Calculation of population indices

For testing whether genetic parameters of the populations studied significantly reflect mean differences in cellular and biochemical heat stress response, three population indices were calculated from the COI dataset. They comprised within-site (‘diversity’) and between-site (‘divergence’) parameters.

The first parameter was nucleotide diversity π (average number of nucleotide differences per site within populations based on the K2P model of sequence evolution), estimated in Arlequin 3.5.1.2 [40].

The two divergence parameters were Nei's [41] pairwise fixation index (F_ST_) and haplotype divergence (H_MH_) based on the Morisita-Horn index [42], both calculated in the R 2.15 statistical environment [43]. For the former index, we used the adegenet package (version 1.3-6) [44]; for the latter index we treated haplotypes as species [45], [46] and estimated the dissimilarity between the haplotype structures of two groups with the vegan package (version 1.17–7) [47].

#### Correlation of physiological heat stress response and genetic data

In order to test whether genetic snail parameters, in principle, are correlated with physiological heat stress responses, we performed Multivariate Regression Tree (MRT) analyses [48]. This multivariate statistics was specifically designed to assess relationships between species information and environmental data. According to the original author, the method is particularly suited to analyze complex environmental information that includes imbalanced and missing information, and non-linear relationships among variables [48]. Using this method, the genetic indices estimated above were assessed for their predictive value to discriminate splits in hierarchical dichotomous clustering of physiological heat stress response data (i.e., Hsp70 and histological data). The clusters and their dependence on the physiological data are graphically represented by a tree. MRT's are not based on traditional significance testing but on 10-fold cross-validation (CV) in order to determine the number of nodes and the importance of predictor variables [48]. Accordingly, the selected genetic indices maximize the homogeneity of measures of stress response within groups of populations and this separation is consistent even if 10% of the populations are omitted.

As it is not possible to use between-population indices (i.e., H_MH_ and F_ST_) for explaining within-population heat stress responses, we performed classical multidimensional scaling (PCoA) with the coordinates of each population assigned to the ordination axes [48], [49]. Hsp70 and histological data were assembled according to populations. Then we constructed individual MRTs for each set of temperature specific heat stress response data (i.e., 25, 33, 40, and 48°C) with the mvpart 1.6-0 package [50] in R 2.15 and 1000 CV. Dependent physiological variables were mean Hsp70 levels and mean assessment values for tubules, calcium cells, and digestive cells; genetic explanatory variables consisted of π and PCoA transformed divergence parameters H_MH_1-H_MH_3 (3 axes), and F_ST_1/F_ST_2 (2 axes).

## Results

### Histopathology

#### Qualitative assessment

The observed reactions in the hepatopancreas were qualitatively similar in all seven populations. Hence, the results can be summarized as follows.

**Figure 1:**
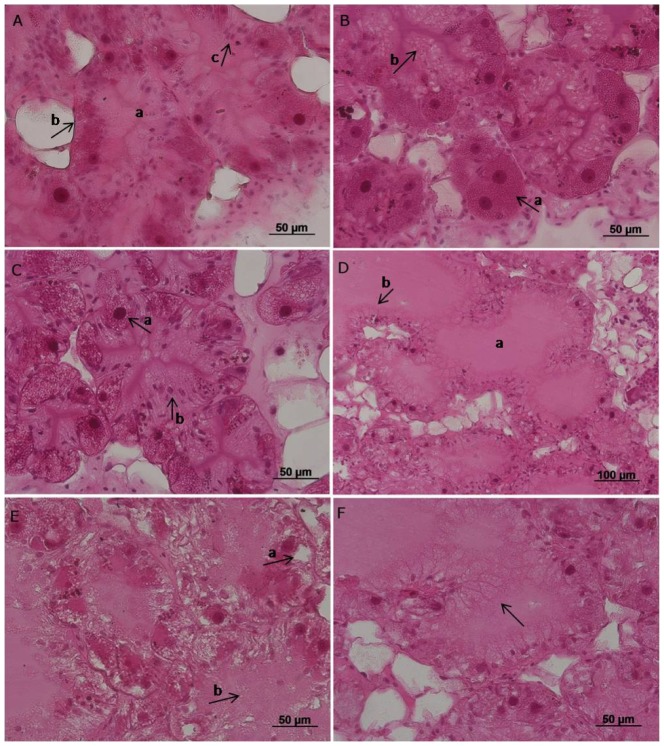
Digestive gland of *Xeropicta derbentina* in different reaction states. **A.** Digestive gland of a control animal. a. indicates tight lumina, b. a smooth base of the tubule and c. shows an oval-shaped nucleus and regular vacuolization of the digestive cells. **B.** Digestive gland of a control animal. a. shows a calcium cell with dense cytoplasm and round nucleus. b. indicates an irregular vacuolization of the digestive cells with partially fused vacuoles. **C.** Digestive gland in state of reaction. a. indicates dark nuclei and an irregular cytoplasm of the calcium cells. Also hypertrophy of the calcium cells occurs. **D.** Digestive gland in state of reaction. a. shows enlarged lumina of the tubule and b. shows pronounced and ruptured apices of the digestive cells. **E.** Digestive gland in state of destruction. a. indicates a very irregular cytoplasm with bright spots in the calcium cells. b. shows cell fragments in the lumen of the tubule. Cell borders are disengaged. **F.** Digestive gland in state of destruction showing necrosis. The arrow indicates ruptured cell apices. Cell borders are disengaged.

*Tubules*: In the control group, the lumina of the tubules were narrow and the cellular bases and apices appeared relatively smooth. After exposure to higher temperatures, tubules of the digestive gland showed enlarged lumina with ruptured apices and also irregular bases primarily caused by hypertrophic calcium cells (Fig. 1D, F). Cell fragments, notably of the digestive cells, could be found in the lumina after 48°C exposure (Fig. 1F).

*Digestive cells*: The digestive cells showed a regular vacuolization and compartmentation and an oval-shaped nucleus in the control group (Fig. 1A). Following elevated temperature levels, we found incidence of an irregular cellular compartmentation and vacuolization with enhanced and partially fused vacuoles (Fig. 1B). The cell apices appeared convex, protruded into the lumen of the tubules, and were often ruptured (Fig. 1B, D, F). Deformation and enlargement of the nuclei could be detected. Especially in the groups exposed to higher temperature, the cell apices were ruptured, cell borders were disengaged, and nuclei damaged (Fig. 1D, F). Both cell lysis and necrosis increasingly occurred in the 48°C group (Fig. 1F).

*Calcium cells*: In the control group, calcium cells showed a dense cytoplasm and spherical nuclei (Fig. 1B). With elevated temperature, reduced density of the cytoplasm with bright spots, disturbed compartmentation, and an increasing vacuolization could be observed (Fig. 1C, E). Furthermore, the nuclei of the calcium cells were either enlarged or deformed with reduced size and, additionally, appeared dark. Cell shape was altered and, in few cases, hypertrophy of the cells could be detected (Fig. 1C). Necrosis could be observed at 48°C (Fig. 1F).

#### Semi-quantitative assessment

Within all populations, the structural symptoms observed in the temperature-exposed test groups and categorized as described above were compared to the control group (25°C).

**Figure 2:**
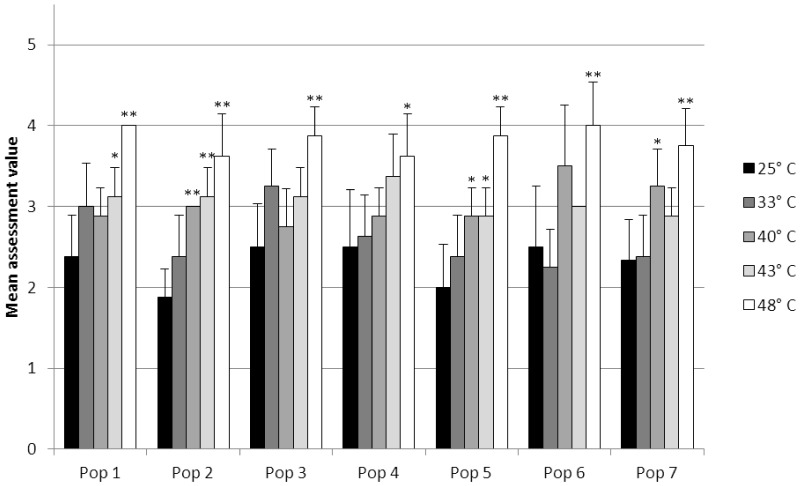
The structural condition of the hepatopancreatic tubules. Mean assessment values for each population at elevated temperature. Shown are means and SD; *n* = 8. Asterisks show significant differences of the respective exposure groups compared to the control at 25°C after Bonferroni correction: 0.0025<*P*≤0.0125: (*) and 0.00025<*P*≤0.0025 (**).

The integrity of the hepatopancreatic tubules became significantly different from the control status after exposure to 40°C (population 2), 43°C (population 2), and 48°C (populations 1, 2, 3, 5, 6 and 7). Slightly significant differences from the control status occurred at 40°C (populations 5 and 7), 43°C (populations 1 and 5), and 48°C (population 4) (Fig. 2).

**Figure 3:**
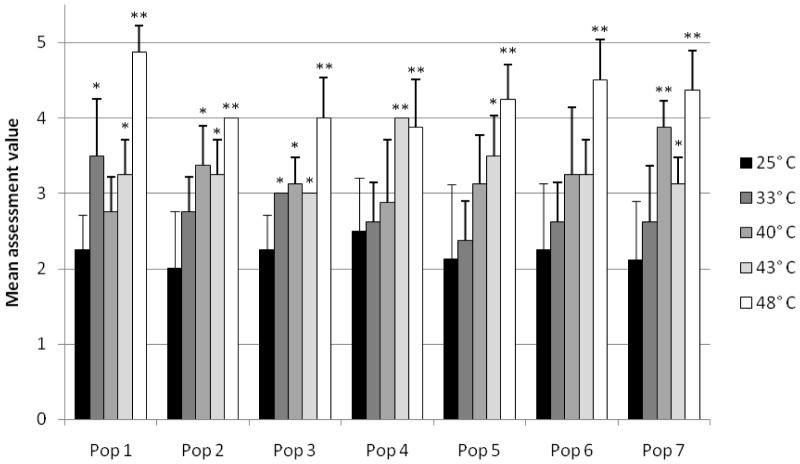
The condition of the digestive cells of the hepatopancreas. Mean assessment values for each population at elevated temperature. Shown are means and SD; *n* = 8. Asterisks show significant differences of the respective exposure groups compared to the control at 25°C after Bonferroni correction: 0.0025<*P*≤0.0125: (*) and 0.00025<*P*≤0.0025 (**).

The digestive cells reacted in a significantly different way from the control group after exposure to 48°C in all populations. Already slightly significant differences were detected after exposure to 33°C (populations 1 and 3), 40°C (populations 2 and 3), and 43°C (populations 1, 2, 3 and 7). Population 7 showed a significant impairment after exposure to 40°C or 48°C, and a slightly significant deterioration of the digestive cells in the 43°C group (Fig. 3).

**Figure 4:**
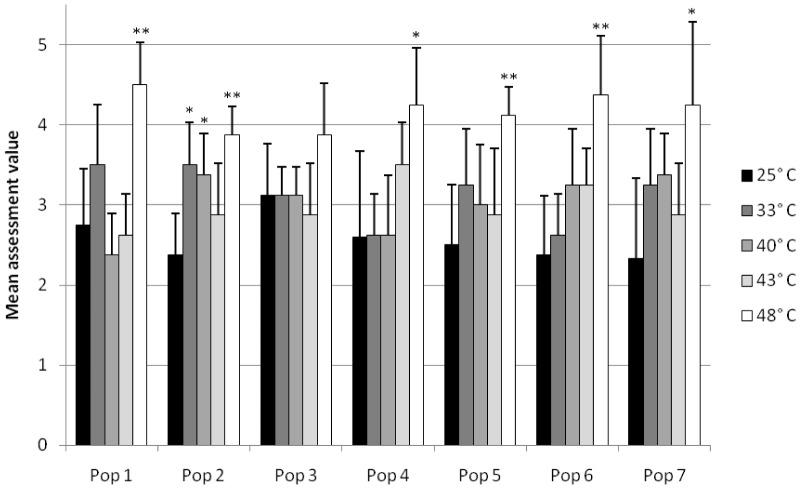
The condition of the calcium cells of the hepatopancreas. Mean assessment values for each population at elevated temperature. Shown are means and SD; *n* = 8. Asterisks show significant differences of the respective exposure groups compared to the control at 25°C after Bonferroni correction: 0.0025<*P*≤0.0125: (*) and 0.00025<*P*≤0.0025 (**).

The calcium cells displayed slightly significant reactions after exposure to 48°C (populations 4 and 7). Population 2 exhibited a slightly significant impairment already after 33 and 40°C exposure and a significant difference from the control status after exposure to 48°C. Also populations 1, 5, and 6 showed first significant deterioration of the calcium cells after exposure to 48°C. Population 3 did not show any significant alterations to the control, even under high temperature regimes, but the calcium cells were already in a ‘status of reaction’ in the control group (Fig. 4).

#### Percentage of calcium cells

No significant increase in the ratio of calcium cells in the digestive gland could be detected at elevated temperature levels in any of the studied populations. In comparison to the results of Dittbrenner et al. [27] who observed a gradual increase of calcium cells after exposure to different temperature regimes in two populations of *X. derbentina*, our investigated populations already showed a rather high number of calcium cells (around 40–50% of all digestive gland cells) in the control group (25°C) and were not able to increase this ratio significantly at high temperatures.

#### Ratio of digestive cell and calcium cell integrity

**Figure 5:**
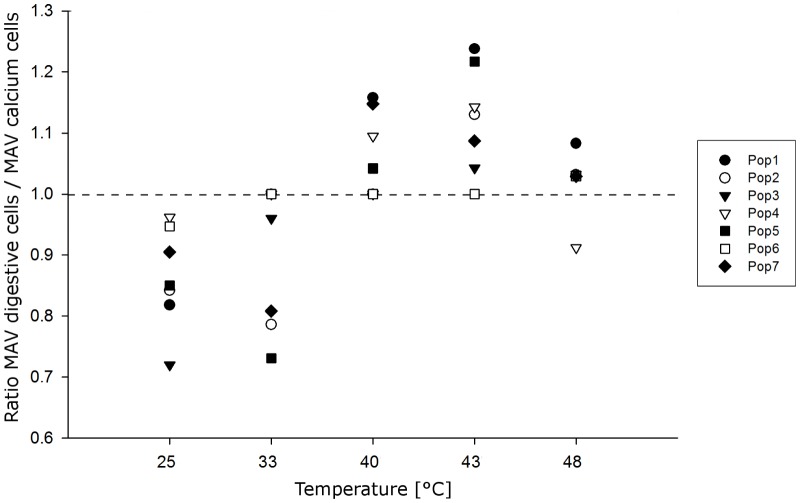
Ratio of the mean assessment values obtained for digestive cells and calcium cells. The ratio for each population at different temperature regimes is illustrated. Data below the dashed line indicate a better condition of digestive cells, compared to calcium cells. Data above the line indicate the opposite.

Digestive cells and calcium cells showed different modes of reaction at elevated temperature in the investigated populations. A ratio of the integrity of the digestive cells and the integrity of the calcium cells was calculated for each population and exposure group, illustrated in Fig. 5. In general, digestive cells remained in a better health condition than the calcium cells at lower temperatures (25 and 33°C). With elevated temperature, however, the digestive cells became more deteriorated than the calcium cells. Populations 1–5, and 7 showed a continuously increasing deterioration of the digestive cells, compared to the condition of the calcium cells, up to exposure to 43°C (or 40°C, population 7). In population 6, however, the condition of the digestive cells was slightly ‘better’ than the condition of the calcium cells in the 25°C group, but following elevating temperature, the integrity of these two cell types was rather equal. For the 48°C exposure, the assessment values of both cell types generally became relatively equal caused by degradation of either cell type. In population 4, however, even at this high temperature, the digestive cells were in a slightly ‘better’ condition than the calcium cells.

### Stress protein analyses

Our pre-test on a possible relevance of shell size on stress protein expression did not reveal any significant effect of shell size on Hsp70 expression neither on the Hsp70 base level nor on the Hsp70 levels recorded after temperature exposure.

The actual stress protein analyses indicated that almost all populations showed an up-regulation of their stress protein level until 40°C followed by a decrease of Hsp70 values at higher temperatures. However, populations 2, 3, 4, and 7 already exhibited a high base level of Hsp70 at control temperature, and populations 2 and 4 were not able to raise their Hsp70 levels remarkably.

**Figure 6:**
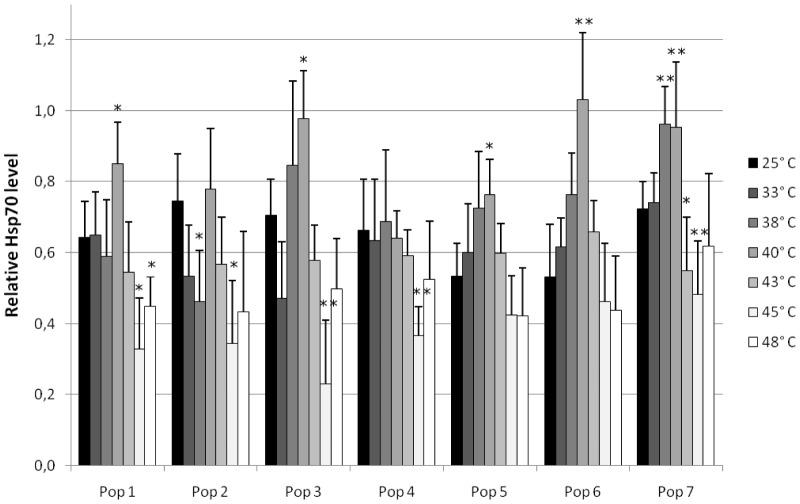
Relative Hsp70 level of different populations after exposure to elevated temperature for 8 h. Shown are means and SD; *n* = 10. Asterisks show significant differences of the respective exposure groups compared to the control at 25°C after Bonferroni correction: 0.0017<*P*≤0.0083: (*) and 0.00017<*P*≤0.0017 (**).

To test for significant differences, exposure groups were compared to the control group (25°C) within each population. A slightly significant increase of the Hsp70 level could be detected at 40°C (populations 1, 3, 5, and 7). In population 7, we detected a significant increase in the Hsp70 level after exposure to 38 and 40°C. Also, population 6 showed a significant increase of stress proteins at 40°C exposure. In the high temperature group of 45°C, the decrease of the stress protein level was slightly significant (populations 1 and 2) or significant (populations 3, 4 and 7) compared to the control group. Population 2 already showed a slightly significant decrease at 38°C (Fig. 6). In addition, a slightly significant decrease was observed after exposure to 43°C (population 7) and 48°C (population 1).

All populations showed a maximum induction of Hsp70 at 40°C, except for populations 4 and 7, which peaked in their Hsp70 level at 38°C. Population 6 revealed the highest maximum stress protein induction with 193.9%, whereas population 4 was not able to increase its stress protein level appreciably (104.2%). Population 3 also showed a relatively high maximum induction followed by population 5 and 7. A rather low maximum stress protein induction was detected in population 1 and 2 (Table 3).

**Table 3:** Maximum levels of Hsp70 induction in different populations after exposure to elevated temperature regimes.

Population	Temperature	Maximum Hsp70 induction
1	40°C	132.3%
2	40°C	125.7%
3	40°C	155.9%
4	38°C	104.2%
5	40°C	142.8%
6	40°C	193.9%
7	38°C	140.0%

The maximum induction was calculated as the ratio vs. the Hsp70 induction at the control temperature (25°C).

**Figure 7:**
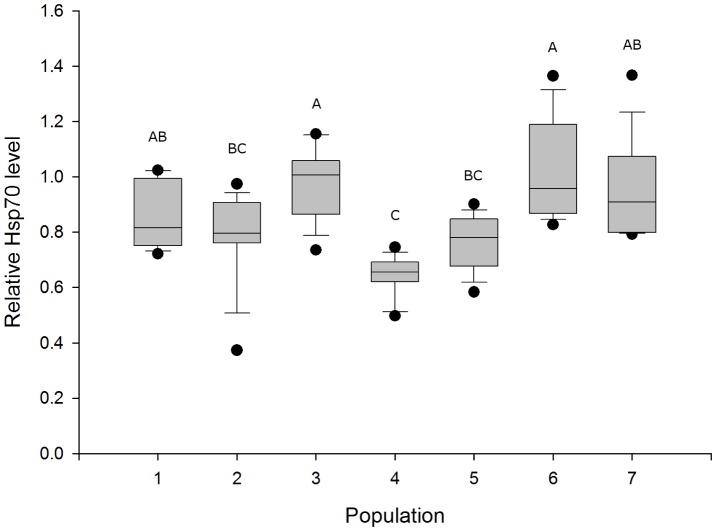
Comparison of Hsp70 levels after exposure to 40°C among populations. Different letters indicate statistically significant differences between groups. Data is plotted as the median, 10th, 25th, 75th, and 90th percentiles. Points show minimum and maximum values.

In general, maximal Hsp70 induction was observed in the 40°C exposure group (38°C not histopathologically analyzed). Therefore, we compared Hsp70 data of this group with those of the other populations. Our analysis of variance showed differences among groups (F_6,63_ = 9.1987; p<0.0001). Population 4 was significantly different from population 1 (p = 0.0291), population 3 (p<0.0001), population 6 (p<0.0001), and population 7 (p = 0.0002). Population 3 was significantly different from populations 2 (p = 0.0472) and 5 (p = 0.0246). Population 6, too, showed a significant difference from populations 2 (p = 0.0043) and 5 (p = 0.002) (Fig. 7). For details see Table S1.

### Correlation of Hsp70 level and histopathology

**Figure 8:**
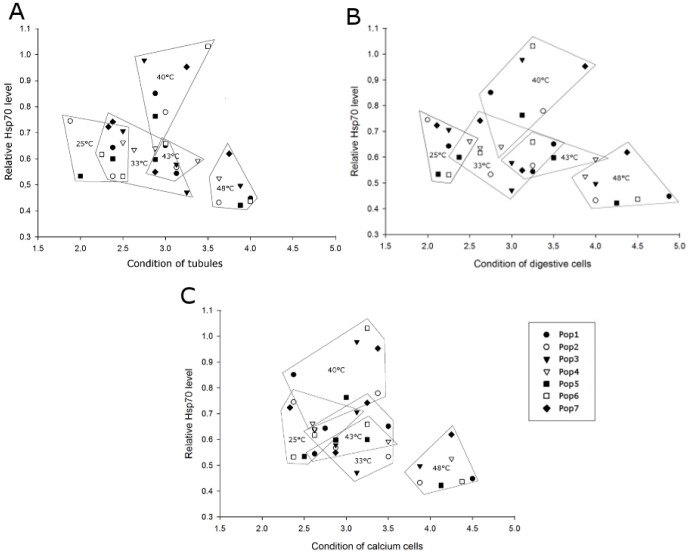
Correlation of relative Hsp70 level vs. histopathological mean assessment values. Data obtained for the populations of the respective exposure groups (25, 33, 40, 43 and 48°C) are framed, respectively. **A.** Relative Hsp70 level vs. condition of the tubules. **B.** Relative Hsp70 level vs. condition of the digestive cells. **C.** Relative Hsp70 level vs. condition of the calcium cells.

The relative Hsp70 level was plotted against the histopathological assessment of the tubule structure, the digestive cells, and the calcium cells, respectively (Fig. 8). Generally, the Hsp70 level increased up to its maximum at 40°C along with increasing cellular responses (MAV 2.5–3.5), and decreased in parallel to further increasing cellular injury with rising temperature. However, the respective populations showed different patterns in respect to this correlation: populations 3, 6, and 7 reached a high stress protein level at 40°C, whereas the other populations, especially population 4, kept their stress protein levels relatively low. Despite their high Hsp70 levels, populations 6 and 7 showed stronger cellular alterations than the other ones. Population 4, however, already revealed distinct cell damages at 43°C exposure. A conspicuous improvement of the cellular condition after stress protein level elevation could be observed in population 1.

The differences in temperature stress-response patterns between the studied populations can be further described as follows. Generally, all populations showed a good cellular condition in the control group of 25°C for all assessed parameters (condition of tubule structure, digestive cells and calcium cells) which went along with low (populations 1, 4, 5, and 6) or intermediate Hsp70 levels (populations 2, 3, and 7). Only individuals of population 3 already revealed their calcium cells to be in the ‘status of reaction’ in response to 25°C.

Population 1 showed, along with an increasing Hsp70 level, distinct improvements in cellular condition. The cells of this population were in the ‘status of reaction’ already after exposure to 33°C (accompanied by a low stress protein level), but a conspicuous improvement of cellular integrity associated with an increase in Hsp70 level occurred after 40°C heat exposure. Digestive and calcium cells revealed an improved histological picture in which the calcium cells were even in ‘better’ condition than those of the control group at 25°C.

Despite of high Hsp70 base levels, population 3 was able to increase its stress protein induction to rather high levels at 40°C exposure. In the 33°C group, we detected a low Hsp70 level associated with general cellular reactions. After increasing the Hsp70 level (40°C), the tubule structure improved whereas the condition of digestive and calcium cells stayed in the same category (status of reaction). Contrary to population 1, population 3 did not show any improvement in the structure of digestive and calcium cells in the company of high Hsp70 levels. Though, individuals of this population where, in spite of changes in the Hsp70 level, able to keep the status of digestive cells (up to 43°C) and calcium cells (up to 40°C) relatively constant (MAV around 3.0). Here, the cellular integrity of the hepatopancreas seemed to be rather independent from Hsp70.

In contrast, population 4 kept its stress protein level on a relatively low level in all exposure groups. This was nevertheless associated with a good cellular condition until 40°C. However, only this population showed prominent cellular deterioration after exposure to 43°C - especially the condition of the digestive cells obviously declined - whereas all other populations revealed better cellular condition at this temperature.

Populations 6 and 7 were able to raise their stress protein level significantly after 40°C exposure, but exhibited cellular reactions in all assessment groups (all assessed parameters were in ‘status of reaction’). Especially population 7 showed strongly damaged digestive cells.

Populations 2 and 5 revealed a relatively low Hsp70 level at elevated temperatures. In spite of a slightly increase of stress proteins in the 40°C group, histological reactions could be observed particularly in the digestive and the calcium cells of population 2. Population 5 showed increasing deterioration of the cellular status with elevated temperature regimes, whereas, accompanied by an increase in stress proteins, the cellular condition was at a moderate level at 40°C.

Despite of a decrease of Hsp70 in all populations after exposure to 43°C for 8 h, we observed, compared to the 40°C exposure, a general structural improvement of cells in population 7. Compared to 40°C, also a ‘better’ condition in digestive cells (populations 2 and 3) and calcium cells (populations 2, 3, and 5) was detected at 43°C. Population 1 still showed a reasonably ‘good’ condition of the calcium cells at 43°C (MAV 2.6), which differed only marginally from the cell status at 40°C.

In the 48°C exposure group, the cellular condition declined from the maximum hand in hand with a decrease of the Hsp70 in all populations. Conspicuously, population 7 showed a higher Hsp70 level compared to the other populations, and population 2 revealed totally damaged digestive cells (MAV 4.8).

As mentioned before, digestive and calcium cells showed divergent modes of reaction when subjected to elevated temperature regimes. In general, the digestive cells were in ‘better’ condition than the calcium cells after exposure to 25 and 33°C, and the stress protein levels stayed on a low or moderate level. After an increase of Hsp70 induction in the 40°C group, the calcium cells revealed a ‘better’ status compared to the condition of the digestive cells (populations 1, 4, 5, and 7), or both cell types were in rather equal conditions (populations 2, 3, and 6). After exposure to 43°C, the condition of the calcium cells was generally ‘better’ than the condition of the digestive cells in all populations, accompanied by a decrease in the stress protein level.

### Genetic analyses

#### Network analysis

The statistical parsimony analysis of the 700 base pair long COI fragment of 138 individuals resulted in a single parsimonious network with a total of 6 haplotypes (network not shown here). The majority of the sequences belonged to two haplotypes (66% and 19% of all sequences), which were present in all populations. More than 99% of nucleotide positions were shared among haplotypes, strongly suggesting that all individuals belong to the same species.

#### Population indices

Genetic diversity within populations (π) was generally low (all<0.004). The highest diversity was found in populations 4 and 1; population 7 was homogeneous (Table 4). Genetic differences among populations, expressed by F_ST_ and H_MH_, were also relatively low (Table 4). The only exception was population 4, which showed relatively high values for both F_ST_ and H_MH_ and thus is most dissimilar to all other *X. derbentina* populations studied.

**Table 4:** Within- and between-site genetic differentiation calculated for *Xeropicta derbentina* populations (1–7) from Southern France based on the COI gene.

	1	2	3	4	5	6	7
1	0.0032±0.0018	0.05	0.13	0.75	0.01	0.10	0.21
2	0.030	0.0024±0.0015	0.03	0.77	0.09	0.01	0.07
3	0.073	0.021	0.0023±0.0014	0.79	0.21	0.03	0.06
4	0.287	0.338	0.315	0.0036±0.0020	0.75	0.77	0.80
5	0.005	0.056	0.115	0.316	0.0030±0.0017	0.14	0.27
6	0.068	0.010	0.0328	0.407	0.100	0.0016±0.0011	0.03
7	0.235	0.117	0.108	0.551	0.290	0.088	0

On diagonal line: nucleotide diversity (π); above diagonal: haplotype divergence (H_MH_) based on the Morisita-Horn index; below diagonal: pairwise fixation index (F_ST_).

#### Correlation of physiological heat stress response and genetic data

The results of the individual MRT analyses under different temperature settings are provided in Table 5 (group-specific trees given in Newick format). Under all temperature conditions, physiological data are well explained by genetic variables (particularly divergence parameters) as indicated by R^2^ ranging from 50–78% (CV errors 1.03–2.95). The highest R^2^ was observed under the 40°C condition (i.e., the condition under which the populations showed the highest Hsp70 activities). There, a divergence parameter (F_ST_) could explain the physiological parameters that led to the primary grouping (i.e., the first split) of the tree.

**Table 5:** Results of the MRT analyses of PCoA transformed physiological heat stress response data (Hsp70 and histology) constrained with population structure information of *Xeropicta derbentina* under four temperature conditions.

Temperature setting	R^2^ (CV-error)	Tree topology of populations (Newick format)	Explanatory variables for primary grouping	Correlation of explanatory and dependent variables for primary grouping
25°C	50% (1.31)	(1,3,4),(2,5,6,7)	H_MH_3	Hsp70 (+), histology (d)
33°C	58% (1.25)	(2,4,5,6,7),(1,3)	H_MH_3	Hsp70 (+), histology (i)
40°C	78% (1.03)	((1,4),(3,5)),(2,6,7)	F_ST_1	Hsp70 (−), histology (i)
48°C	50% (2.95)	(1,5,6),(2,3,4,7)	F_ST_2	Hsp70 (+), histology (i)

R^2^: cross-validated proportion of variance explained by the primary grouping (i.e., first split of the tree); P1–P7: populations studied; π: nucleotide diversity; H_MH_3: axis 3 of transformed haplotype diversity; F_ST_1, F_ST_2: axes 1 and 2 of transformed pairwise fixation index; (+): positive correlation; (−): negative correlation; improved histopathology (i); deteriorated histopathology (d).

At 33°C, the proportion of variance explained by the first split of the tree was 58% with the physiological parameters being explicated by the divergence parameter H_MH_. Both at 25°C and 48°C, R^2^ for the first split of the trees was 50% with the physiological parameters being explained by divergence indices (H_MH_ and F_ST_).

Interestingly, in the 25, 33, and 48°C groups divergence parameters are positively correlated with Hsp70 levels (i.e., specimens that show high genetic differentiations to specimens from neighboring populations are characterized by high HSP70 levels), whereas for the 40°C condition, the correlation is inversely. In contrast, high divergence values are associated with adverse histopathological effects under 25°C conditions, whereas under 33, 40, and 48°C conditions, specimens that show high genetic differentiations to specimens from neighboring populations typically show fewer adverse effects.

Cross-validations produced large errors, which can be explained by the relatively small number of populations studied.

## Discussion

Heat stress affects organisms at different physiological levels, including biochemical defense reactions mirrored by the cellular status of central metabolic organs as, e.g., the hepatopancreas in mollusks. The hepatopancreas is strongly involved in metabolic processes even under normal conditions [9], [10], [51]. Besides other factors, the metabolic rate can increase due to high temperatures [52] and, thereby, increases the need of nutrient supply by the hepatopancreas. As found in the qualitative assessment of the hepatopancreatic tubule structure, the lumina of the tubules were dilated after exposure to higher temperatures, which could have been the result of an increased metabolic rate associated to a demand in nutrient supply.

An increased amount of ruptured cell apices primarily of the digestive cells occurred preferentially after exposure to higher temperatures, which could be explained by an activated release of lysosomal enzymes. Lysosomal membranes are known to disintegrate under elevated temperatures [53], which cause lysosomal enzymes to be released. Poste et al. [54] showed that an extracellular release of these enzymes from damaged lysosomes can destroy cell membranes and generally alter cell structures. The combination of high temperature and low pH (also as a result of high temperature) enhance the disruption of lysosomal membranes and also the activity of released enzymes [53].

Predominantly after exposure to high temperature, the calcium cells showed dark nuclei which are indicative of a low pH [55], disturbed compartmentation, and reduced density of the cytoplasm. Calcium cells play an important role in osmoregulation [10] and the acid-base balance [13]. It is known that heat can negatively affect the acid-base balance [56] and the ion-balance as a result of water-loss by increased evaporation which leads to osmotic stress and acidosis. High temperatures can also lower the pH [57], which leads to an accumulation of acidic metabolic products in snail tissue, causing metabolic acidosis [58]. Related to these facts, we assume that the observed heat effects in the calcium cells are associated with osmotic stress and a disturbed acid-base balance. Occasionally, we observed calcium cells exhibiting some heat stress symptoms in the control group (population 3). This might be due to the fact that these snails had already encountered high temperatures in the field and did not fully recover during acclimatization time in the lab prior to the experiments. Scheil et al. [29] also observed cellular reactions in the control group of a *X. derbentina* population after acclimation and concluded that this might have been caused by pre-exposure in the field.

Our results showed digestive cells to be more heat sensitive than calcium cells. In almost all populations, the condition of the digestive cells stayed ‘below’ the status of reaction at temperatures of up to 33°C, as illustrated in Fig. 5. With rising temperature, they showed irregular vacuolization and fused vacuoles, both being indicative for an active cellular response, and ruptured cell apices which are likely due to the release of lysosomal enzymes as mentioned above. A main function of the digestive cells is resorption of nutrients and intracellular digestion. Because the two lowest temperatures correspond to natural environmental conditions allowing these snails to be active and feed, it becomes reasonable that their metabolism ensures a stable function of this cell type at these temperatures. When temperatures rise, e.g. in the morning of hot summer days [31], the snails remain attached inactive on vegetation. During this period, they are fully exposed to the sun, heat up, and need to cope with heat stress. The above-mentioned functions of the calcium cells become more important under these conditions, so we can assume that under these circumstances, a functional status of these cells is of higher importance than that of the digestive cells.

In several studies, an increase in the percentage of the extension of calcium cells in the digestive gland, caused by hyperplasia, hypertrophy, or loss of digestive cells as an adaptation to heat stress, was observed [27], [59]. In this study, the ratio of calcium cells did not differ among the treatment groups in any of the investigated populations. Only in few cases, hypertrophy of calcium cells could be detected, and also no decrease in the number of digestive cells was observed. However, the populations investigated in this study already revealed a rather high percentage of calcium cells (about 40–50%) in the control group, so we assume that snails were not able to raise this level remarkably in response to heat. Also in a study by Scheil et al. [29], a high percentage of calcium cells in controls and only a minor increase in their surface ratio were observed when *X. derbentina* was exposed to 45°C.

In order to better understand these histological findings, it is necessary to compare the histopathological results to those obtained for stress proteins.

In response to increasing temperatures, all populations showed an up-regulation of Hsp70 up to a distinct level. This maximum was followed by a decrease in stress protein level as a result of exposure to higher temperatures. These findings are in accordance with the kinetics of stress protein induction described by Eckwert et al. [60]. The induction of Hsp70 is known to be due to proteotoxic effects of stressors in cells and the subsequent initiation of stress gene transcription (compensation phase). After reaching the maximum level of stress protein induction, the stress response decreases, presumably caused in most cases by a pathological impairment of the stress protein machinery (destruction phase). In our study, the histopathological data confirm this interpretation, particularly for temperatures >40°C.

**Figure 9:**
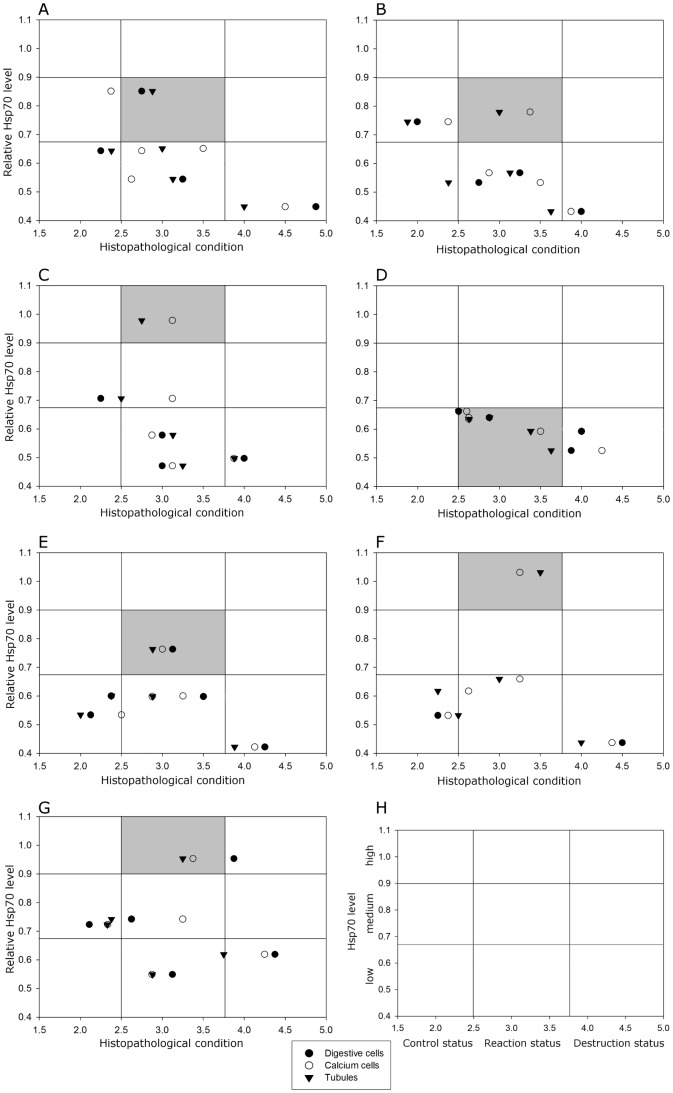
Correlation of relative Hsp70 levels vs. mean histopathological assessment values for each population. Shaded squares reflect the respective population's strategy of Hsp70 induction at the histological ‘status of reaction’ of the hepatopancreas. The position of vertical and horizontal lines is arbitrary and for visual purposes only. **A.** Population 1 **B.** Population 2 **C.** Population 3 **D.** Population 4 **E.** Population 5 **F.** Population 6 **G.** Population 7 **H.** Explanation of subdivisions of Hsp70 levels and histopathological conditions as applied to plots A–G.

It is known that land snails living under extreme environmental conditions and suffering from heat overload and desiccation use Hsp induction as important survival strategy [26], [61]. Furthermore, the expression of Hsp70 proteins is thought to be very energy costly [62]–[64]. Differences among species and populations in the intensity in which stress proteins are induced could be associated with differences among them in how temperature has affected their energy budgets [65]. With respect to the relative Hsp70 levels (in association with the cellular mean assessment values) in our study which predominantly differed at 40°C among the populations (Fig. 7 and 8), the investigated populations follow different strategies to arrange with thermal stress:

Strategy 1: investment in medium Hsp70 levels and keeping cellular condition on the level of moderate response (populations 1, 2, and 5), strategy 2: spending energy in high Hsp70 levels associated with cellular condition on a moderate level (populations 3, 6, and 7), and strategy 3: no investment in significantly elevated Hsp70 levels, but nevertheless insurance of cellular functionality until a certain temperature, at the risk of a rapid cellular decay at extreme temperature (population 4). These strategies are reflected in Fig. 9, which illustrates the association of Hsp70 level and histopathological condition for each population. The distribution of Hsp70 maxima and statistical analyses displayed in Fig. 7 support our grouping of the snail populations in these strategies.

What could be the benefits of the strategies involving either high or low levels of Hsp70? Due to the energy consumption associated with the synthesis of Hsps and the expense related to the synthesis of other types of proteins at elevated temperature levels [65], over-expression of Hsps can reduce fitness [66]–[68]. Consequently, lower levels of Hsp70 might save fitness costs. Another aspect is that in evolution thermal resistance or tolerance to chemical stressors often is achieved in other ways presumably due to the costs of the production of high Hsp70 levels [64], [69], [70]. Mizrahi et al. [26] demonstrated that a Mediterranean snail species (*Sphincterochila cariosa*), which is rather sensitive to desiccation, showed a higher level of Hsp72 compared to a related, desert-inhabiting snail species (*Sphincterochila zonata*) that is more desiccation resistant. They also suggested that these results are in line with other studies demonstrating higher levels of Hsp70 in heat sensitive species compared to heat tolerant ones [24], [71]. Assuming that low Hsp70 levels are indicative of phenotypes that have been selected for thermotolerance, one could conclude that populations 6 and 7 of our study did not exhibit this tolerance as they showed cellular decay despite high Hsp70 levels.

Because each population has its own demographic history (e.g., bottlenecks, immigration, emigration), it is not surprising, that Hsp70 expression levels are specimen and population specific. In fact, previous population- or line-specific analyses of Hsp70 expression levels in other invertebrates clearly demonstrated within and among population differences in Hsp70 levels [22]–[24]. However, the specific demographic parameters responsible for the correlation of population structure and Hsp70 levels are still poorly understood. We therefore tested several candidate explanatory genetic parameters in this study in order to infer their effects on physiological heat stress response data. Note, however, that the mitochondrial COI gene used in this study can only reflect the phylogeographical and demographic history of our populations studied; it is very likely not directly involved in Hsp70 expression.

Interestingly, our MRT analyses (Table 5) showed that physiological data are well explained by genetic population characteristics in general and by divergence parameters in particular. This is especially evident in the 40°C exposure group (R^2^ = 78%), the condition with the maximal stress protein induction. Here, little genetic differentiation between populations is associated with high Hsp70 levels and improved histopathological conditions. In other words, specimens that share haplotypes with specimens from neighboring populations show, on average, higher Hsp70 values and fewer adverse histopathological effects. The overall pattern inferred could possibly be explained by regional population processes that are particularly acting at 40°C, that is, an ecologically realistic, yet the highest non-lethal temperature. In contrast, under the other temperature regimes tested (i.e., 25, 33, and 48°C), local processes appear to be more important.

Why do some populations not show cellular deterioration even though their Hsp70 level remained low after 8 h exposure to thermal stress? Scheil et al. [29] showed that Hsp70 was up-regulated already after 0.5 h of exposure time, reaching a significant peak after 4 h and then decreased, reaching the base level again after 8 h. In addition, they observed no significant impact on the integrity in cellular condition during exposure time. Thus, it cannot be excluded that short-time induction of stress proteins, which may have occurred within the first hours of exposure (but remain undetected after 8 h), protected cells from pathology.

It is particularly striking that digestive cells and calcium cells showed different modes of reaction in response to the tested gradient of elevated temperature in the investigated populations. This variation in reaction patterns of digestive and calcium cells was found to be associated with the Hsp70 level: with increasing stress protein content, the condition of calcium cells improved or, at least, did not decline in all populations. We conclude that Hsp70 has a protective effect especially on the calcium cells and that our studied populations invested energy to ensure the function of this cell type, above all because of its important role in osmoregulation [10] and acid-base balance [13]. This observation can also be linked to the function of calcium cells in protein synthesis [9], [10]. Up to now, there are no studies about the intensity of Hsp70 synthesis in calcium cells of the hepatopancreas in snails. It is, however, reasonable to assume that Hsp70 synthesis took place in the calcium cells, and that an increase in stress protein synthesis could lead to an instant protection effect in this cell type. This topic might be addressed by further studies in the future.

Our study showed that there is considerable variation in the survival strategies in populations of *X. derbentina*. Results indicate that populations invest either more or less energy in elevated Hsp70 synthesis, according to the presumed trade-off with fitness costs. We observed populations that at least invested energy in moderately elevated (population 1, 2, and 5) or high stress protein levels (population 3, 6, and 7) to keep cellular condition stable. Furthermore, one population (population 4), was able to keep cellular functionality despite a low, at most slightly elevated, Hsp70 level until 40°C exposure, whereas prominent cellular reactions were observed beyond this thermal limit in this population only. Generally, we observed that, with an elevation in Hsp70 levels, especially after exposure to high temperature, calcium cells seemed to be more heat tolerant than digestive cells.

Genetic analyses showed that physiological data are well explained by genetic variables, especially for the 40°C exposure group. This possibly indicates strong selective pressures acting at this high, but environmentally relevant temperature. Despite the presumable uniformity in the requirements for the survival of different *X. derbentina* populations at high temperature, our study nevertheless showed the cellular components of survival strategies of snail populations to be very variable. In concert with one another, these components are apparently equally efficient in different *X. derbentina* populations and enable survival of them in their natural habitats.

## Supporting Information

Table S1 Results of the Tukey Kramer HSD post-hoc test for the comparison of Hsp70 levels after 40°C exposure among populations (p-values are shown).
(DOCX)
