# Supplementary material for: Intraspecific Variation in Cellular and Biochemical Heat Response Strategies of Mediterranean Xeropicta derbentina [Pulmonata, Hygromiidae]
Source: PLoS One. 2014 Jan 27;9(1):e86613. doi: 10.1371/journal.pone.0086613 (PMC3903566; doi:10.1371/journal.pone.0086613)
Supplement: Table S1 — Results of the Tukey Kramer HSD post-hoc test for the comparison of Hsp70 levels after 40°C exposure among populations (p-values are shown). (DOCX) [file pone.0086613.s001.docx]

**Table S1.** Results of the Tukey Kramer HSD post-hoc test for the comparison of Hsp70 levels after 40°C exposure among populations (p-values are shown).

|  | Population 1 | Population 2 | Population 3 | Population 4 | Population 5 | Population 6 | Population 7 |
| --- | --- | --- | --- | --- | --- | --- | --- |
| Population 1 | * |  |  |  |  |  |  |
| Population 2 | 0,9213 | * |  |  |  |  |  |
| Population 3 | 0,4538 | 0,0472 | * |  |  |  |  |
| Population 4 | 0,0291 | 0,3458 | <0,0001 | * |  |  |  |
| Population 5 | 0,8224 | 1,000 | 0,0246 | 0,4925 | * |  |  |
| Population 6 | 0,0958 | 0,0043 | 0,9812 | <0,0001 | 0,0020 | * |  |
| Population 7 | 0,7028 | 0,1198 | 0,9997 | 0,0002 | 0,0679 | 0,8867 | * |
